# Structural and electronic data of three first-row transition octahedral hexaaquametal(II) ions, metal=Cr, Ni or Cu

**DOI:** 10.1016/j.dib.2018.11.055

**Published:** 2018-11-15

**Authors:** Jeanet Conradie

**Affiliations:** Department of Chemistry, University of the Free State, PO Box 339, Bloemfontein 9300, South Africa

## Abstract

Structural and density functional theory calculated data of three octahedral hexaaquametal(II) ions containing different metals (Cr, Ni or Cu) are presented to illustrate different geometries these octahedral hexaaquametal(II) ions can have. The density functional theory optimized geometries exhibit either a regular octahedral geometry, an octahedral elongated or an octahedral compressed geometry. Experimental structures exhibit octahedral or distorted octahedral geometries, the latter includes octahedral elongated, octahedral compressed and orthorhombic distorted geometries.

**Specifications table**

**Table:** 

Subject area	*Chemistry*
More specific subject area	*Computational chemistry*
Type of data	*Table, graph, figure*
How data were acquired	*High performance computing (HPC) facility.*
Data format	*Analyzed.*
Experimental factors	*Experimental data were obtained from the Cambridge Structural Data Centre.*
Experimental features	*DFT data were obtained with the Gaussian 09 program on the High Performance Computing facility of the University of the Free State.*
Data source location	*Department of Chemistry, University of the Free State, Nelson Mandela street, Bloemfontein, South Africa.*
Data accessibility	*Data are with article.*
Related research article	*J. Conradie, Jahn–Teller effect in high spin d*^*4*^*and d*^*9*^*octahedral metal-complexes, Inorg. Chim Acta, 486 (2019) 193–199*[1].

**Value of the data**

• This data provide density functional theory calculated optimized geometries of regular octahedral, octahedral elongated and octahedral compressed hexaaquametal(II) complexes.
• This data can be used to visualize, illustrate and investigate the electronic structure of octahedral, octahedral elongated and octahedral compressed hexaaquametal(II) complexes.
• Example input files and coordinates can be used for DFT calculations.
• This data can be used to visualize the influence of degenerate electronic ground states of hexaaquametal(II) molecules on the geometry of the molecule.
• The data can be used to illustrate the Jahn–Teller effect on hexaaquametal(II) molecules exhibiting degenerate molecular energy levels.

## 1 Data

**Fig. 1:**
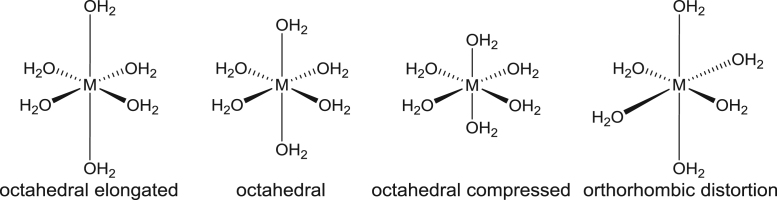
Structures of octahedral hexaaquametal(II) complexes.

**Fig. 2:**
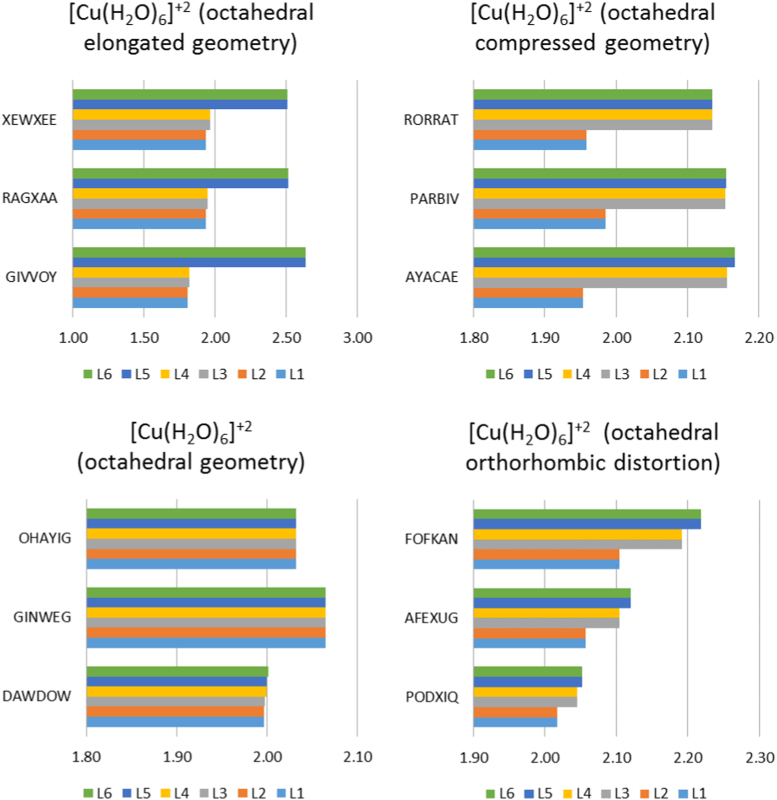
Illustration of Cu─O bond lengths (L1–L6 in Å on *x*-axis) for different experimentally observed geometries of selected [Cu(OH_2_)_6_]^2+^ ions. The CSD reference code is indicated [2].

**Fig. 3:**
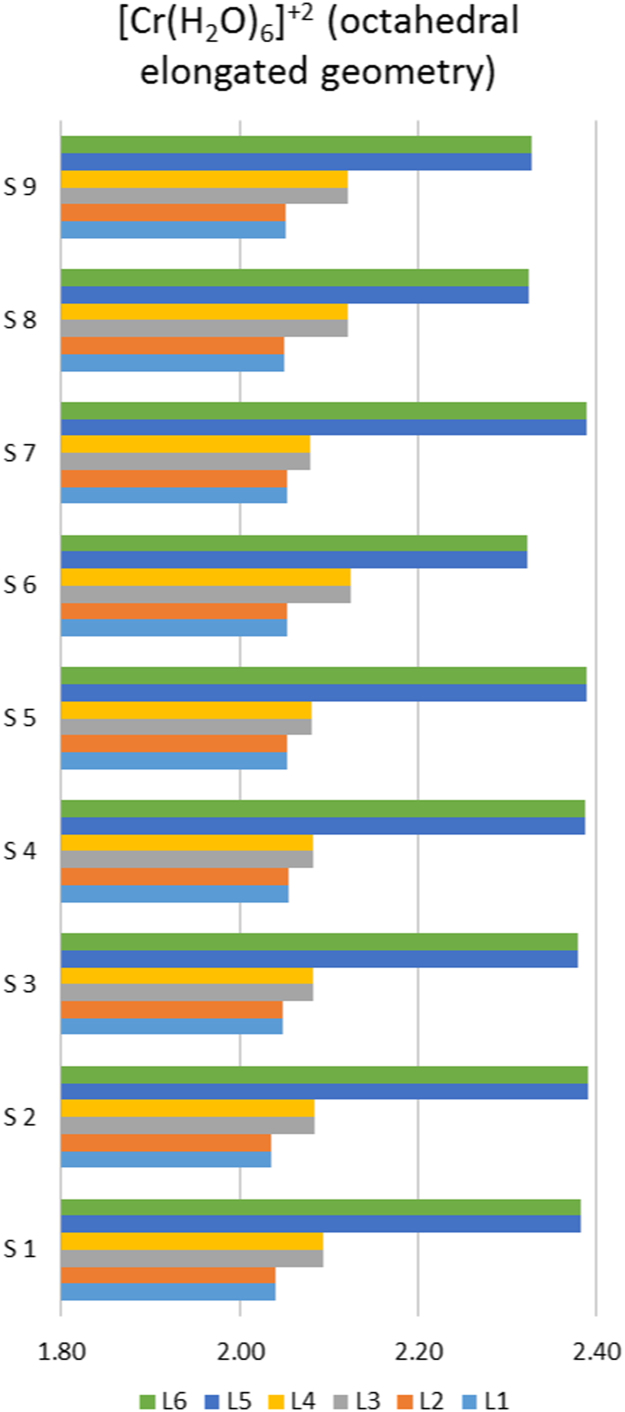
Illustration of Cr─O bond lengths (L1–L6 in Å on *x*-axis) for experimentally observed octahedral elongated geometries of selected [Cr(OH_2_)_6_]^2+^ ions. Data from references [3] for S1–S4, [4] for S5, [5] for S6 and S7, [6] for S8 and [7] for S9.

Density functional theory calculated data of the M–O bonds of optimized octahedral hexaaquametal(II) ions containing different metals (Cr, Ni or Cu), are given in Table 1 and the respective coordinates of the different structures in Supporting information. The density functional theory (DFT) optimized geometries exhibit either a regular octahedral geometry, an octahedral elongated or an octahedral compressed geometry (see Fig. 1). The triplet of the hexaaquanickel(II) complex ion, [Ni(OH_2_)_6_]^2+^, with d-electron occupation $d_{\mathrm{xy}}^{2}d_{\mathrm{xz}}^{2}d_{\mathrm{yz}}^{2}d_{z^{2}}^{1}d_{{x^{2}-y}^{2}}^{1}$, without any degenerate electronic ground states optimized to an octahedral geometry with all the Ni–O bonds of the same length, see data in Table 1. The quintet of the hexaaquachromium(II) complex ion, [Cr(OH_2_)_6_]^2+^, optimized to two electronic states namely $d_{\mathrm{xy}}^{1}d_{\mathrm{xz}}^{1}d_{\mathrm{yz}}^{1}d_{z^{2}}^{1}d_{{x^{2}-y}^{2}}^{0}$ and $d_{\mathrm{xy}}^{1}d_{\mathrm{xz}}^{1}d_{\mathrm{yz}}^{1}d_{{x^{2}-y}^{2}}^{1}d_{z^{2}}^{0}$, an octahedral elongated and an octahedral compressed geometry respectively, see data in Table 1. Similarly the doublet of the hexaaquacopper(II) complex ion, [Cu(OH_2_)_6_]^2+^, optimized to the electronic states $d_{\mathrm{xy}}^{2}d_{\mathrm{xz}}^{2}d_{\mathrm{yz}}^{2}d_{z^{2}}^{2}d_{{x^{2}-y}^{2}}^{1}$ and $d_{\mathrm{xy}}^{2}d_{\mathrm{xz}}^{2}d_{\mathrm{yz}}^{2}d_{{x^{2}-y}^{2}}^{2}d_{z^{2}}^{1}$, with an octahedral elongated and an octahedral compressed geometry respectively, see data in Table 1. The DFT calculated data compare well with available experimental data. A summary of available experimental structural data for selected hexaaquametal(II) ions is given in Supporting information Table S1 for [Cu(OH_2_)_6_]^2+^ [2], Table S2 for [Cr(OH_2_)_6_]^2+^ [3], [4], [5], [6], [7], [8] and Table S3 for [Ni(OH_2_)_6_]^2+^ [2]. Selected experimental structural data of [Cu(OH_2_)_6_]^2+^, illustrated in Fig. 2, show that the experimental geometry of [Cu(OH_2_)_6_]^2+^ can be octahedral, octahedral elongated, octahedral compressed or orthorhombic distorted, though the preferred structure of [Cu(OH_2_)_6_]^2+^ is octahedral elongated, see structural data in Table S1. Crystallographic data (Table S2) as well as EPR studies showed that [Cr(OH_2_)_6_]^2+^ preferably exhibit an octahedral elongated complex [9]. (Fig. 3).

**Table 1:** DFT calculated average metal─O bond lengths (Å) for the indicated complexes by the indicated functional and the 6–311G(d,p) basis set.

Complex	Occupation	d(M–L)_*z*-axis ave_	d(M–L)_*xy*-plane ave_	d(M–L)_*z*-axis ave_ - d(M–L)_*xy*-plane ave_	Geometry	DFT functional
[Ni(OH_2_)_6_]^2+^	$d_{\mathrm{xy}}^{2}d_{\mathrm{xz}}^{2}d_{\mathrm{yz}}^{2}d_{z^{2}}^{1}d_{{x^{2}-y}^{2}}^{1}$	2.067	2.067	0.00	Octahedral	B3LYP
		2.051	2.051	0.00		M06
		2.100	2.100	0.00		OLYP
		2.055	2.055	0.00		BP86
		2.00–2.14	2.00–2.14			Exp.
[Cr(OH_2_)_6_]^2+^	$d_{\mathrm{xy}}^{1}d_{\mathrm{xz}}^{1}d_{\mathrm{yz}}^{1}d_{z^{2}}^{1}d_{{x^{2}-y}^{2}}^{0}$	2.343	2.102	0.24	Octahedral elongated	B3LYP
		2.293	2.076	0.22		M06
		2.425	2.123	0.30		OLYP
		2.352	2.082	0.27		BP86
		2.32–2.39	2.04–2.13			Exp.
	$d_{\mathrm{xy}}^{1}d_{\mathrm{xz}}^{1}d_{\mathrm{yz}}^{1}d_{{x^{2}-y}^{2}}^{1}d_{z^{2}}^{0}$	2.046	2.197	−0.15	Octahedral compressed	M06
[Cu(OH_2_)_6_]^2+^	$d_{\mathrm{xy}}^{2}d_{\mathrm{xz}}^{2}d_{\mathrm{yz}}^{2}d_{z^{2}}^{2}d_{{x^{2}-y}^{2}}^{1}$	2.247	2.007	0.24	Octahedral elongated	B3LYP
		2.248	2.008	0.24		M06
		2.350	2.043	0.31		OLYP
		2.254	2.004	0.25		BP86
		2.16–2.64	1.81–2.09			Exp.
	$d_{\mathrm{xy}}^{2}d_{\mathrm{xz}}^{2}d_{\mathrm{yz}}^{2}d_{{x^{2}-y}^{2}}^{2}d_{z^{2}}^{1}$	1.956	2.119	−0.16	Octahedral compressed	M06
		1.95–1.99	2.14–2.17			Exp.

**Fig. 4:**
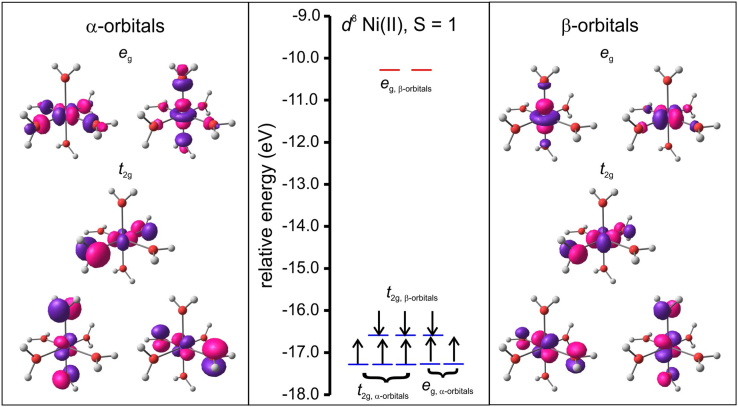
Molecular energy level diagram showing selected energy levels *e*_g_ and *t*_2g_, of the B3LYP/6–311G(d,p) optimized triplet of hexaaquanickel(II) complex ion, [Ni(OH_2_)_6_]^2+^, in a regular octahedral environment. The Ni-based anti-bonding molecular orbitals are also shown. The energy levels of filled MOs are shown in blue, and of empty MOs in red. The arrows indicate the *α*-electrons (up spin) and *β* electrons (down spin). A *T*_h_ symmetry restrained geometry was used to construct the diagram.

**Fig. 5:**
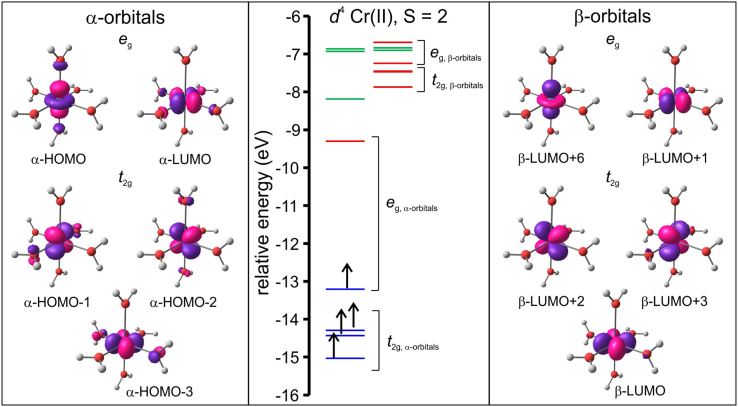
Molecular energy level diagram, including selected molecular orbitals (MOs), of the frontier MO energy levels of the B3LYP/6–311G(d,p) optimized quintet of the hexaaquachromium(II) complex ion, [Cr(OH_2_)_6_]^2+^, showing elongation Jahn–Teller distortion. The energy levels of filled *d*-based MOs are shown in blue, and of empty *d*-based MOs in red. MOs without significant metal-*d* character are shown in green. The arrows indicate the α-electrons (up spin). A *D*_2_ symmetry restrained geometry was used to construct the diagram.

**Fig. 6:**
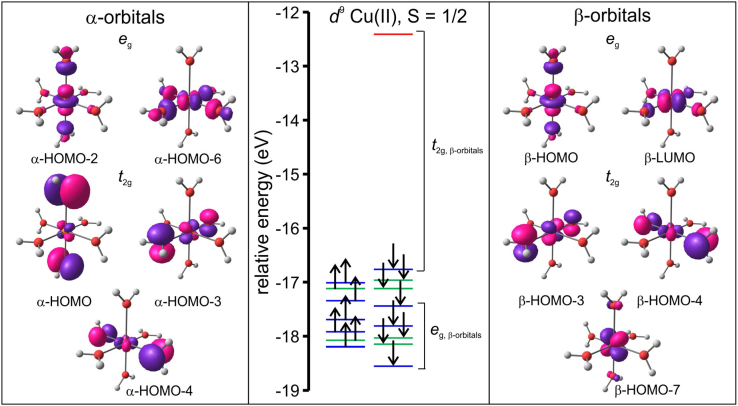
Molecular energy level diagram, including selected molecular orbitals, of the B3LYP/6–311G(d,p) optimized doublet of hexaaquacopper(II) complex ion, [Cu(OH_2_)_6_]^2+^, showing elongation Jahn–Teller distortion. The energy levels of filled *d*-based MOs are shown in blue, and of empty *d*-based MOs in red. MOs without significant metal-*d* character are shown in green. The arrows indicate the α-electrons (up spin). A *D*_2_ symmetry restrained geometry was used to construct the diagram.

The data visualized in Fig. 4 show that the molecular orbital energy levels of the B3LYP/6–311G(d,p) optimized triplet of the hexaaquanickel(II) complex ion, [Ni(OH_2_)_6_]^2+^, in an octahedral environment, without any Jahn–Teller distortion is grouped into the *e*_g_ and *t*_2g_ groups. The data visualized in Figs. 5 and 6 illustrate the split of the molecular orbital energy levels of the B3LYP/6–311G(d,p) optimized quintet of [Cr(OH_2_)_6_]^2+^ and [Cu(OH_2_)_6_]^2+^ respectively, with elongation Jahn–Teller distortion [10]. The metal-d based molecular orbitals (MOs) of [Ni(OH_2_)_6_]^2+^, [Cr(OH_2_)_6_]^2+^ and [Cu(OH_2_)_6_]^2+^ is also visualized in Fig. 4, Fig. 5, Fig. 6. The highest occupied molecular orbital (HOMO) of both [Cr(OH_2_)_6_]^2+^ and [Cu(OH_2_)_6_]^2+^ exhibits $d_{z^{2}}$ character. The anti-bonding between the $d_{z^{2}}$ orbital on the metal and the *p*_*z*_ orbital on the oxygens along the *z*-axis results in elongation Jahn–Teller distortion, i.e. elongation of the M–O bond lengths in the *z*-axis direction; compared to the M–O bonds in the *xy*-plane for $d_{\mathrm{xy}}^{1}d_{\mathrm{xz}}^{1}d_{\mathrm{yz}}^{1}d_{z^{2}}^{1}d_{{x^{2}-y}^{2}}^{0}$ [Cr(OH_2_)_6_]^2+^ and $d_{\mathrm{xy}}^{2}d_{\mathrm{xz}}^{2}d_{\mathrm{yz}}^{2}d_{z^{2}}^{2}d_{{x^{2}-y}^{2}}^{1}$ [Cu(OH_2_)_6_]^2+^, but not for $d_{\mathrm{xy}}^{2}d_{\mathrm{xz}}^{2}d_{\mathrm{yz}}^{2}d_{z^{2}}^{1}d_{{x^{2}-y}^{2}}^{1}$ [Ni(OH_2_)_6_]^2+^.

## 2 Experimental design, materials, and methods

The hexaaquametal(II) ions were optimized with the density functional theory (DFT) computational chemistry program Gaussian 09 [11] using different functionals such as B3LYP [12], [13], BP86 [14], [15], OLYP [13], [16], [17], [18] and M06 [19] in combination with the triple-ζ basis set 6–311G(d,p). Input coordinates (without any symmetry) for the different hexaaquametal(II) ions were constructed using ChemCraft [20]. Input coordinates for symmetry constrained optimizations were obtained from the *C*_1_ optimized coordinates, using the “edit, set point group” option in Chemcraft, to change the coordinates to the desired symmetry. A frequency analysis was performed on all optimized geometries to confirm that these structures correspond to minima on ground state potential energy surfaces. Example input files for the DFT calculations, output files and the optimized Cartesian coordinates are provided in Supporting Information.
