# Supplementary material for: Structural and electronic data of three first-row transition octahedral hexaaquametal(II) ions, metal=Cr, Ni or Cu
Source: Data Brief. 2018 Nov 15;21:2051–8. doi: 10.1016/j.dib.2018.11.055 (PMC6262161; doi:10.1016/j.dib.2018.11.055)
Supplement: Supplementary file 1 — Supplementary material [file mmc1.docx]

The author declare that there is no conflict of interest regarding the publication of this article
