# Supplementary material for: Structural and electronic data of three first-row transition octahedral hexaaquametal(II) ions, metal=Cr, Ni or Cu
Source: Data Brief. 2018 Nov 15;21:2051–8. doi: 10.1016/j.dib.2018.11.055 (PMC6262161; doi:10.1016/j.dib.2018.11.055)
Supplement: Supplementary file 2 — Supplementary material [file mmc2.docx]

**Electronic structure of octahedral hexaaquametal(II) complexes, metal = Ni, Cr or Cu**

Jeanet Conradie*

Department of Chemistry, PO Box 339, University of the Free State, 9300 Bloemfontein, Republic of South Africa.

Supporting information

Additional tables

Table S 1. Experimental Cu─O bond lengths (L1 – L6 in Å) for [Cu(OH_2_)_6_]^2+^ ions as obtained from the CSD [^[[1]](#endnote-1)^].

| CSD code | L1 | L2 | L3 | L4 | L5 | L6 |
| --- | --- | --- | --- | --- | --- | --- |
| **octahedral elongated** | | | | | | |
| AQOXIO | 1.948 | 1.948 | 1.979 | 1.979 | 2.380 | 2.380 |
| BENSCU | 1.956 | 1.956 | 1.979 | 1.979 | 2.260 | 2.260 |
| BENSCU | 1.962 | 1.962 | 1.985 | 1.985 | 2.264 | 2.264 |
| CUMALH01 | 1.949 | 1.949 | 1.993 | 1.993 | 2.408 | 2.408 |
| CUMALH02 | 1.950 | 1.950 | 1.999 | 1.999 | 2.406 | 2.406 |
| DEVMAT | 1.967 | 1.975 | 1.986 | 1.987 | 2.350 | 2.410 |
| DEVMAT01 | 1.973 | 1.973 | 1.980 | 1.980 | 2.395 | 2.395 |
| DEVMAT02 | 1.966 | 1.966 | 1.974 | 1.974 | 2.384 | 2.384 |
| DILCIL | 1.942 | 1.942 | 1.965 | 1.965 | 2.384 | 2.384 |
| DIXGIB | 1.974 | 1.974 | 2.030 | 2.030 | 2.301 | 2.301 |
| DODDOP | 1.939 | 1.939 | 1.997 | 1.997 | 2.412 | 2.412 |
| EBIKOR | 1.973 | 1.973 | 1.991 | 1.991 | 2.397 | 2.397 |
| FECQEK | 2.027 | 2.037 | 2.069 | 2.087 | 2.162 | 2.166 |
| FINNUM | 1.968 | 1.968 | 1.972 | 1.972 | 2.283 | 2.283 |
| GACJEB | 1.956 | 1.956 | 1.959 | 1.959 | 2.350 | 2.350 |
| GIVVIS | 1.933 | 1.933 | 1.945 | 1.945 | 2.458 | 2.458 |
| GIVVOY | 1.809 | 1.809 | 1.819 | 1.819 | 2.641 | 2.641 |
| IBUHUJ | 1.967 | 1.983 | 1.984 | 1.985 | 2.283 | 2.341 |
| KADFIG | 1.990 | 1.990 | 2.004 | 2.004 | 2.406 | 2.406 |
| KAGNEM | 1.953 | 1.953 | 1.960 | 1.960 | 2.418 | 2.418 |
| KAYWAI | 1.925 | 1.925 | 1.991 | 1.991 | 2.332 | 2.332 |
| NAYGUR | 1.958 | 1.975 | 1.985 | 1.999 | 2.295 | 2.304 |
| NERKUQ | 1.968 | 1.968 | 1.968 | 1.968 | 2.376 | 2.376 |
| NINVOW | 1.941 | 1.941 | 1.977 | 1.977 | 2.506 | 2.506 |
| RAGXAA | 1.938 | 1.938 | 1.948 | 1.948 | 2.515 | 2.515 |
| SAFLAM01 | 1.957 | 1.957 | 1.972 | 1.972 | 2.321 | 2.321 |
| SIYZIJ | 1.945 | 1.945 | 1.953 | 1.953 | 2.416 | 2.416 |
| TOLSCV | 1.953 | 1.953 | 1.954 | 1.954 | 2.423 | 2.423 |
| VAFKIX | 1.962 | 1.962 | 1.992 | 1.992 | 2.324 | 2.324 |
| WIHLAC | 1.969 | 1.969 | 1.974 | 1.974 | 2.310 | 2.310 |
| WULSAY | 1.964 | 1.975 | 1.989 | 1.989 | 2.375 | 2.383 |
| WULSAY01 | 1.965 | 1.965 | 1.973 | 1.973 | 2.387 | 2.387 |
| XENHUV | 1.964 | 1.974 | 1.995 | 2.014 | 2.300 | 2.352 |
| XENJAD | 1.964 | 1.968 | 1.992 | 2.005 | 2.297 | 2.346 |
| XEWXEE | 1.938 | 1.938 | 1.968 | 1.968 | 2.509 | 2.509 |
| YEDJUM | 1.951 | 1.951 | 1.968 | 1.968 | 2.385 | 2.385 |
| ZOPFOB | 1.962 | 1.962 | 1.972 | 1.972 | 2.389 | 2.389 |
| SARGAW | 1.968 | 1.968 | 1.970 | 1.970 | 2.372 | 2.372 |
| JADPEJ | 1.947 | 1.947 | 1.985 | 1.985 | 2.382 | 2.382 |
| NULDII | 1.938 | 1.938 | 2.009 | 2.009 | 2.360 | 2.360 |
| PIVJIO | 1.916 | 1.927 | 2.024 | 2.037 | 2.284 | 2.406 |
| UGOZAT | 1.935 | 1.935 | 2.016 | 2.016 | 2.314 | 2.314 |
| VAFKIX | 1.962 | 1.965 | 2.004 | 2.013 | 2.230 | 2.327 |
| VURHOH | 1.958 | 1.958 | 2.006 | 2.006 | 2.356 | 2.356 |
| WUQQAC | 1.935 | 1.935 | 1.979 | 1.979 | 2.398 | 2.398 |
| KARHOD | 1.946 | 1.946 | 2.013 | 2.013 | 2.270 | 2.270 |
| UVOQON | 1.955 | 1.955 | 2.003 | 2.003 | 2.355 | 2.355 |
| AMATUF | 1.978 | 1.978 | 2.036 | 2.036 | 2.248 | 2.248 |
| **octahedral compressed** | | | | | | |
| AYACAE | 1.953 | 1.953 | 2.155 | 2.155 | 2.166 | 2.166 |
| PARBIV | 1.985 | 1.985 | 2.153 | 2.153 | 2.154 | 2.154 |
| RORRAT | 1.959 | 1.959 | 2.135 | 2.135 | 2.135 | 2.135 |
| **octahedral** | | | | | | |
| DAWDOW | 1.997 | 1.997 | 1.998 | 2.000 | 2.000 | 2.001 |
| GINWEG | 2.065 | 2.065 | 2.065 | 2.065 | 2.065 | 2.065 |
| OHAYIG | 2.032 | 2.032 | 2.032 | 2.032 | 2.032 | 2.032 |
| **orthorhombic distortion** | | | | | | |
| AFEXUG | 2.057 | 2.057 | 2.105 | 2.105 | 2.120 | 2.120 |
| IFICUW | 2.038 | 2.048 | 2.051 | 2.056 | 2.062 | 2.070 |
| MAQDOY | 2.072 | 2.072 | 2.073 | 2.104 | 2.104 | 2.105 |
| MOLDAU | 2.019 | 2.019 | 2.022 | 2.022 | 2.076 | 2.076 |
| PODXIQ | 2.017 | 2.017 | 2.045 | 2.045 | 2.053 | 2.053 |
| QEYNAL | 2.022 | 2.022 | 2.089 | 2.089 | 2.108 | 2.108 |
| XUXSIT | 2.093 | 2.093 | 2.104 | 2.104 | 2.112 | 2.112 |
| YEDZOX | 2.040 | 2.070 | 2.080 | 2.098 | 2.111 | 2.139 |
| PIVJOU | 2.055 | 2.075 | 2.086 | 2.189 | 2.203 | 2.293 |
| SOFRIP | 2.023 | 2.023 | 2.064 | 2.064 | 2.066 | 2.066 |
| FOFKAN | 2.104 | 2.104 | 2.192 | 2.192 | 2.219 | 2.219 |

Table S 2. Experimental Cr─O bond lengths (L1 – L6 in Å) for [Cr(OH_2_)_6_]^2+^ ions as obtained literature.

|  | L1 | L2 | L3 | L4 | L5 | L6 | Temperature | Reference |
| --- | --- | --- | --- | --- | --- | --- | --- | --- |
| **octahedral elongated** | | | | | | | | |
| S 1 | 2.04 | 2.04 | 2.094 | 2.094 | 2.382 | 2.382 | 296 K | ^[[2]](#endnote-2)^ |
| S 2 | 2.035 | 2.035 | 2.084 | 2.084 | 2.391 | 2.391 | 100 K | 2 |
| S 3 | 2.049 | 2.049 | 2.083 | 2.083 | 2.38 | 2.38 | 296 K | 2 |
| S 4 | 2.055 | 2.055 | 2.082 | 2.082 | 2.387 | 2.387 | 100 K | 2 |
| S 5 | 2.054 | 2.054 | 2.0802 | 2.0802 | 2.389 | 2.389 | 4.3 K | ^[[3]](#endnote-3)^ |
| S 6 | 2.054 | 2.054 | 2.125 | 2.125 | 2.323 | 2.323 | 295 K | ^[[4]](#endnote-4)^ |
| S 7 | 2.0532 | 2.0532 | 2.0792 | 2.0792 | 2.3889 | 2.3889 | 84 K | 4 |
| S 8 | 2.0503 | 2.0503 | 2.121 | 2.121 | 2.324 | 2.324 | 295 K | ^[[5]](#endnote-5)^ |
| S 9 | 2.052 | 2.052 | 2.122 | 2.122 | 2.327 | 2.327 | 295 K | ^[[6]](#endnote-6)^ |
| **octahedral** | | | | | | | | |
| S 10 | 2.106 | 2.106 | 2.106 | 2.106 | 2.106 | 2.106 | 293 K | ^[[7]](#endnote-7)^ |

Table S 3. Experimental Ni─O bond lengths (L1 – L6 in Å) for [Ni(OH_2_)_6_]^2+^ ions as obtained from the CSD [1].

| CSD code | L1 | L2 | L3 | L4 | L5 | L6 |
| --- | --- | --- | --- | --- | --- | --- |
| AVOLOO | 2.033 | 2.071 | 2.033 | 2.039 | 2.039 | 2.071 |
| ESIWAG | 2.037 | 2.075 | 2.037 | 2.07 | 2.07 | 2.075 |
| UVUVOY | 2.009 | 2.051 | 2.009 | 2.082 | 2.082 | 2.051 |
| AFEYAN | 2.064 | 2.074 | 2.064 | 2.03 | 2.03 | 2.074 |
| AJIQOZ | 2.047 | 2.064 | 2.047 | 2.044 | 2.044 | 2.064 |
| AROMIE | 2.034 | 2.05 | 2.034 | 2.065 | 2.065 | 2.05 |
| ASAXAU | 2.039 | 2.046 | 2.039 | 2.033 | 2.033 | 2.046 |
| ATUQAH | 2.057 | 2.05 | 2.057 | 2.047 | 2.047 | 2.05 |
| AWELAQ | 2.081 | 2.038 | 2.081 | 2.037 | 2.037 | 2.038 |
| AWELEU | 2.066 | 2.074 | 2.066 | 2.035 | 2.035 | 2.074 |
| BECRIL | 2.043 | 2.029 | 2.03 | 2.062 | 2.056 | 2.111 |
| BEMXAT | 2.051 | 2.072 | 2.051 | 2.047 | 2.047 | 2.072 |
| BIZJEA | 2.059 | 2.046 | 2.059 | 2.029 | 2.029 | 2.046 |
| BUJHAQ | 2.091 | 2.022 | 2.062 | 2.091 | 2.022 | 2.062 |
| CAKPIP | 2.066 | 2.1 | 2.066 | 2.009 | 2.009 | 2.1 |
| CEJMEJ | 2.072 | 2.072 | 2.072 | 2.072 | 2.035 | 2.035 |
| CICHIG | 2.046 | 2.042 | 2.07 | 2.091 | 2.047 | 2.038 |
| CIVCAN | 2.051 | 2.057 | 2.051 | 2.068 | 2.068 | 2.057 |
| COCXUO | 2.043 | 2.053 | 2.043 | 2.081 | 2.081 | 2.053 |
| CUPBAQ | 2.06 | 2.065 | 2.06 | 2.067 | 2.067 | 2.065 |
| DEZPEF | 2.055 | 2.052 | 2.055 | 2.041 | 2.041 | 2.052 |
| DEZPEF01 | 2.062 | 2.08 | 2.062 | 2.088 | 2.088 | 2.08 |
| DURQOX | 2.088 | 2.024 | 2.088 | 2.054 | 2.054 | 2.024 |
| EGUNOL | 2.07 | 2.051 | 2.01 | 2.026 | 2.097 | 2.081 |
| EJAMIL | 2.052 | 2.033 | 2.052 | 2.099 | 2.099 | 2.033 |
| EKOYOU | 2.059 | 2.059 | 2.059 | 2.05 | 2.05 | 2.059 |
| ENIYUW | 2.063 | 2.025 | 2.063 | 2.048 | 2.048 | 2.025 |
| EQEHEO | 2.068 | 2.083 | 2.068 | 2.106 | 2.106 | 2.083 |
| FAFLUV | 2.045 | 2.084 | 2.045 | 2.07 | 2.07 | 2.084 |
| FAMKIP | 2.075 | 2.032 | 2.075 | 2.057 | 2.057 | 2.032 |
| FEGLUY | 2.029 | 2.065 | 2.029 | 2.048 | 2.048 | 2.065 |
| FEHWUL | 2.054 | 2.043 | 2.054 | 2.042 | 2.042 | 2.043 |
| FIKCUZ | 2.049 | 2.082 | 2.055 | 2.076 | 2.068 | 2.049 |
| FITREF | 2.049 | 2.046 | 2.049 | 2.087 | 2.087 | 2.046 |
| FITREF01 | 2.046 | 2.05 | 2.046 | 2.085 | 2.085 | 2.05 |
| FOLZIQ | 2.043 | 2.068 | 2.043 | 2.052 | 2.052 | 2.068 |
| FONDOC | 2.015 | 2.058 | 2.015 | 2.058 | 2.015 | 2.015 |
| FONDOC01 | 2.018 | 2.025 | 2.018 | 2.099 | 2.099 | 2.025 |
| GINWAC | 2.051 | 2.051 | 2.051 | 2.051 | 2.051 | 2.051 |
| HEKDIK | 2.041 | 2.061 | 2.066 | 2.007 | 2.043 | 2.058 |
| HEYBET | 2.043 | 2.058 | 2.043 | 2.03 | 2.03 | 2.058 |
| HULMEG | 2.056 | 2.11 | 2.056 | 2.025 | 2.025 | 2.11 |
| IDAGIF | 2.064 | 2.056 | 2.064 | 2.064 | 2.064 | 2.056 |
| IDAPOT | 2.074 | 2.019 | 2.074 | 2.071 | 2.019 | 2.071 |
| IDUSEG | 2.063 | 2.033 | 2.063 | 2.059 | 2.059 | 2.033 |
| IHEQET | 2.052 | 2.042 | 2.027 | 2.038 | 2.013 | 2.053 |
| IMIQAW | 2.077 | 2.077 | 2.009 | 2.077 | 2.077 | 2.009 |
| INAZEC | 2.035 | 2.056 | 2.035 | 2.061 | 2.068 | 2.058 |
| INAZEC | 2.069 | 2.019 | 2.052 | 2.02 | 2 | 2.05 |
| ITUTEW | 2.071 | 2.053 | 2.039 | 2.042 | 2.057 | 2.066 |
| IXOROD | 2.13 | 2.127 | 2.122 | 2.107 | 2.12 | 2.138 |
| IYUKIW | 2.049 | 2.06 | 2.049 | 2.05 | 2.05 | 2.06 |
| IYUKOC | 2.002 | 2.072 | 2.002 | 2.066 | 2.066 | 2.072 |
| JEQMIC | 2.057 | 2.035 | 2.057 | 2.074 | 2.074 | 2.035 |
| JIRRAE | 2.042 | 2.084 | 2.042 | 2.04 | 2.04 | 2.084 |
| KAGNAI | 2.03 | 2.054 | 2.03 | 2.057 | 2.057 | 2.054 |
| KAXLEB | 2.042 | 2.017 | 2.042 | 2.013 | 2.013 | 2.017 |
| KEMWOQ | 2.061 | 2.051 | 2.048 | 2.053 | 2.05 | 2.054 |
| KEXKAA | 2.036 | 2.043 | 2.036 | 2.067 | 2.067 | 2.043 |
| KEXKAA01 | 2.047 | 2.039 | 2.047 | 2.071 | 2.071 | 2.039 |
| KICJUB | 2.09 | 2.058 | 2.09 | 2.043 | 2.043 | 2.058 |
| KIDMAM | 2.032 | 2.068 | 2.032 | 2.046 | 2.046 | 2.068 |
| KIXLIM | 2.06 | 2.047 | 2.06 | 2.023 | 2.023 | 2.047 |
| LALPIY | 2.064 | 2.027 | 2.064 | 2.037 | 2.037 | 2.027 |
| LEFYEB | 2.068 | 2.058 | 2.068 | 2.022 | 2.022 | 2.058 |
| LERMIF | 2.048 | 2.121 | 2.031 | 2.038 | 2.112 | 2.047 |
| LERMIF01 | 2.038 | 2.114 | 2.038 | 2.042 | 2.042 | 2.114 |
| LIJSOM | 2.041 | 2.057 | 2.049 | 2.029 | 2.056 | 2.072 |
| LIKDIT | 2.062 | 2.115 | 2.062 | 2.039 | 2.039 | 2.115 |
| LOFGUK | 2.044 | 2.044 | 2.068 | 2.068 | 2.055 | 2.055 |
| LOFGUK | 2.069 | 2.069 | 2.042 | 2.042 | 2.048 | 2.048 |
| LOPNAH | 2.081 | 2.067 | 2.081 | 2.001 | 2.001 | 2.067 |
| LOVKUE | 2.077 | 2.046 | 2.077 | 2.02 | 2.02 | 2.046 |
| LUMVAQ | 2.047 | 2.023 | 2.047 | 2.077 | 2.077 | 2.023 |
| MACHOO | 2.016 | 2.068 | 2.016 | 2.074 | 2.074 | 2.068 |
| MAPHER | 2.09 | 2.002 | 2.036 | 2.061 | 2.05 | 2.067 |
| MAXDIA | 2.07 | 2.028 | 2.07 | 2.051 | 2.051 | 2.028 |
| MAXDIA | 2.006 | 2.051 | 2.004 | 2.073 | 2.077 | 2.032 |
| MEYLAD | 2.063 | 2.043 | 2.063 | 2.047 | 2.047 | 2.043 |
| NAKTEA | 2.058 | 2.058 | 2.089 | 2.04 | 2.04 | 2.046 |
| NANPIC | 2.017 | 2.082 | 2.017 | 2.069 | 2.069 | 2.082 |
| NELMUO | 2.051 | 2.118 | 2.051 | 2.049 | 2.049 | 2.118 |
| NIGUAS01 | 2.048 | 2.062 | 2.048 | 2.059 | 2.059 | 2.062 |
| NIHSAY | 2.037 | 2.061 | 2.037 | 2.075 | 2.075 | 2.061 |
| NODKUO | 2.028 | 2.053 | 2.028 | 2.032 | 2.032 | 2.053 |
| NOHHEZ | 2.016 | 2.056 | 2.016 | 2.028 | 2.028 | 2.056 |
| NOWTOK | 2.059 | 2.07 | 2.059 | 2.055 | 2.055 | 2.07 |
| NOWTOK | 2.045 | 2.051 | 2.045 | 2.041 | 2.041 | 2.051 |
| NOXBOR | 2.032 | 2.064 | 2.032 | 2.065 | 2.065 | 2.064 |
| NQXSNI | 2.032 | 2.017 | 2.032 | 2.077 | 2.077 | 2.017 |
| NUHRUE | 2.035 | 2.035 | 2.083 | 2.083 | 2.065 | 2.065 |
| NUMTIY | 2.056 | 2.056 | 2.056 | 2.056 | 2.056 | 2.056 |
| PAHXIF | 2.057 | 2.039 | 2.057 | 2.025 | 2.025 | 2.039 |
| PAHXIF01 | 2.042 | 2.042 | 2.074 | 2.028 | 2.076 | 2.076 |
| PEJVAC | 2.041 | 2.08 | 2.041 | 2.031 | 2.031 | 2.08 |
| PEYDAZ | 2.065 | 2.049 | 2.065 | 2.04 | 2.04 | 2.049 |
| PEYDAZ | 2.031 | 2.069 | 2.031 | 2.039 | 2.039 | 2.069 |
| PIWKIR | 2.057 | 2.012 | 2.057 | 2.089 | 2.089 | 2.012 |
| POPBUT | 2.062 | 2.04 | 2.062 | 2.066 | 2.066 | 2.04 |
| PUSMOH | 2.038 | 2.106 | 2.038 | 2.035 | 2.035 | 2.106 |
| QAFQOF | 2.057 | 2.058 | 2.052 | 2.018 | 2.052 | 2.058 |
| QALVAA | 2.056 | 2.048 | 2.056 | 2.053 | 2.053 | 2.048 |
| QELXEK | 2.036 | 2.036 | 2.036 | 2.036 | 2.036 | 2.036 |
| QIXKUE | 2.073 | 2.023 | 2.073 | 2.017 | 2.017 | 2.023 |
| QOCPAA | 2.058 | 2.073 | 2.056 | 2.049 | 2.083 | 2.043 |
| QOSWEC | 2.062 | 2.052 | 2.062 | 2.033 | 2.033 | 2.052 |
| QUKQUI01 | 2.055 | 2.018 | 2.055 | 2.067 | 2.067 | 2.018 |
| QUKQUI02 | 2.017 | 2.054 | 2.017 | 2.054 | 2.054 | 2.054 |
| QUQGEP | 2.026 | 2.024 | 2.024 | 2.024 | 2.026 | 2.026 |
| QURCEL | 2.027 | 2.042 | 2.027 | 2.026 | 2.026 | 2.042 |
| QURCEL | 2.025 | 2.061 | 2.025 | 2.041 | 2.041 | 2.061 |
| RADZUU | 2.056 | 2.059 | 2.056 | 2.068 | 2.068 | 2.059 |
| RADZUU | 2.058 | 2.064 | 2.058 | 2.067 | 2.067 | 2.064 |
| RENROS | 2.068 | 2.054 | 2.068 | 2.064 | 2.064 | 2.054 |
| RITKOW | 2.058 | 2.039 | 2.058 | 2.07 | 2.07 | 2.039 |
| ROLBOK | 2.071 | 2.053 | 2.044 | 2.042 | 2.061 | 2.044 |
| ROLBOK | 2.054 | 2.046 | 2.048 | 2.036 | 2.066 | 2.051 |
| ROLBOK01 | 2.06 | 2.031 | 2.033 | 2.042 | 2.06 | 2.049 |
| ROLBOK01 | 2.04 | 2.026 | 2.05 | 2.027 | 2.041 | 2.066 |
| ROLBOK01 | 2.022 | 2.039 | 2.022 | 2.063 | 2.063 | 2.039 |
| ROLBOK01 | 2.053 | 2.066 | 2.053 | 2.018 | 2.018 | 2.066 |
| ROLBOK02 | 2.069 | 2.051 | 2.044 | 2.038 | 2.062 | 2.043 |
| ROLBOK02 | 2.056 | 2.044 | 2.045 | 2.04 | 2.066 | 2.057 |
| ROLBOK03 | 2.071 | 2.048 | 2.045 | 2.039 | 2.058 | 2.044 |
| ROLBOK03 | 2.055 | 2.046 | 2.048 | 2.038 | 2.067 | 2.054 |
| ROLBOK04 | 2.072 | 2.046 | 2.045 | 2.04 | 2.058 | 2.044 |
| ROLBOK04 | 2.055 | 2.041 | 2.046 | 2.035 | 2.068 | 2.059 |
| ROLBOK05 | 2.066 | 2.047 | 2.043 | 2.035 | 2.058 | 2.046 |
| ROLBOK05 | 2.053 | 2.042 | 2.043 | 2.038 | 2.067 | 2.055 |
| ROLBOK06 | 2.069 | 2.046 | 2.043 | 2.035 | 2.06 | 2.046 |
| ROLBOK06 | 2.05 | 2.042 | 2.043 | 2.042 | 2.065 | 2.054 |
| ROLBOK07 | 2.068 | 2.045 | 2.046 | 2.04 | 2.056 | 2.044 |
| ROLBOK07 | 2.052 | 2.04 | 2.042 | 2.036 | 2.064 | 2.057 |
| ROLBOK08 | 2.07 | 2.043 | 2.045 | 2.037 | 2.057 | 2.047 |
| ROLBOK08 | 2.057 | 2.047 | 2.045 | 2.036 | 2.067 | 2.05 |
| ROLBOK09 | 2.068 | 2.047 | 2.045 | 2.04 | 2.056 | 2.047 |
| ROLBOK09 | 2.055 | 2.042 | 2.044 | 2.037 | 2.068 | 2.055 |
| ROLBOK10 | 2.068 | 2.045 | 2.045 | 2.038 | 2.061 | 2.049 |
| ROLBOK10 | 2.056 | 2.043 | 2.043 | 2.035 | 2.068 | 2.052 |
| ROLBOK11 | 2.072 | 2.043 | 2.045 | 2.036 | 2.056 | 2.047 |
| ROLBOK11 | 2.05 | 2.042 | 2.046 | 2.034 | 2.068 | 2.055 |
| ROLBOK12 | 2.067 | 2.039 | 2.043 | 2.036 | 2.06 | 2.041 |
| ROLBOK12 | 2.054 | 2.041 | 2.043 | 2.04 | 2.066 | 2.056 |
| ROLBOK13 | 2.067 | 2.037 | 2.046 | 2.04 | 2.057 | 2.045 |
| ROLBOK13 | 2.052 | 2.05 | 2.04 | 2.036 | 2.061 | 2.05 |
| ROLBOK14 | 2.067 | 2.042 | 2.044 | 2.039 | 2.059 | 2.05 |
| ROLBOK14 | 2.058 | 2.045 | 2.041 | 2.041 | 2.065 | 2.056 |
| ROLBOK15 | 2.059 | 2.044 | 2.041 | 2.04 | 2.059 | 2.051 |
| ROLBOK16 | 2.06 | 2.045 | 2.041 | 2.04 | 2.058 | 2.05 |
| ROLBOK17 | 2.062 | 2.045 | 2.039 | 2.04 | 2.058 | 2.048 |
| ROLBOK18 | 2.059 | 2.044 | 2.04 | 2.039 | 2.057 | 2.045 |
| ROLBOK19 | 2.058 | 2.041 | 2.04 | 2.039 | 2.063 | 2.047 |
| ROLBOK20 | 2.062 | 2.041 | 2.041 | 2.04 | 2.058 | 2.048 |
| ROLBOK21 | 2.059 | 2.043 | 2.041 | 2.04 | 2.06 | 2.046 |
| ROLBOK22 | 2.058 | 2.042 | 2.041 | 2.04 | 2.06 | 2.048 |
| ROLBOK23 | 2.058 | 2.041 | 2.036 | 2.038 | 2.062 | 2.045 |
| ROLBOK24 | 2.06 | 2.044 | 2.039 | 2.042 | 2.062 | 2.048 |
| ROLBOK25 | 2.06 | 2.047 | 2.042 | 2.04 | 2.063 | 2.047 |
| ROLBOK26 | 2.068 | 2.048 | 2.038 | 2.041 | 2.06 | 2.045 |
| ROLBOK26 | 2.061 | 2.045 | 2.052 | 2.039 | 2.069 | 2.05 |
| ROLBOK27 | 2.069 | 2.05 | 2.04 | 2.042 | 2.061 | 2.043 |
| ROLBOK27 | 2.059 | 2.046 | 2.052 | 2.038 | 2.069 | 2.054 |
| ROLBOK28 | 2.07 | 2.05 | 2.039 | 2.043 | 2.059 | 2.041 |
| ROLBOK28 | 2.058 | 2.043 | 2.052 | 2.039 | 2.068 | 2.052 |
| ROLBOK29 | 2.071 | 2.052 | 2.041 | 2.04 | 2.06 | 2.041 |
| ROLBOK29 | 2.058 | 2.046 | 2.053 | 2.039 | 2.07 | 2.053 |
| ROLBUQ | 2.052 | 2.042 | 2.052 | 2.054 | 2.054 | 2.042 |
| RORQUM | 2.047 | 2.047 | 2.045 | 2.047 | 2.047 | 2.045 |
| SAHPOH | 2.058 | 2.052 | 2.058 | 2.061 | 2.061 | 2.052 |
| SESSUF | 2.034 | 2.082 | 2.034 | 2.062 | 2.062 | 2.082 |
| SETQUD | 2.103 | 2.027 | 2.103 | 2.05 | 2.05 | 2.027 |
| SIDJUL | 2.09 | 2.089 | 2.089 | 2.089 | 2.089 | 2.09 |
| SOFTUD | 2.065 | 2.055 | 2.065 | 2.061 | 2.061 | 2.055 |
| SOFWIU | 2.028 | 2.043 | 2.028 | 2.05 | 2.05 | 2.043 |
| SOQTOI | 2.055 | 2.055 | 2.055 | 2.055 | 2.055 | 2.055 |
| SOQTOI | 2.049 | 2.044 | 2.049 | 2.041 | 2.041 | 2.044 |
| SUVCAN | 2.021 | 2.038 | 2.021 | 2.04 | 2.04 | 2.038 |
| TAKHIX | 2.045 | 2.058 | 2.045 | 2.021 | 2.021 | 2.058 |
| TAKHIX01 | 2.046 | 2.022 | 2.046 | 2.055 | 2.055 | 2.022 |
| TAKHIX02 | 2.056 | 2.025 | 2.056 | 2.046 | 2.046 | 2.025 |
| TAKHIX03 | 2.044 | 2.056 | 2.044 | 2.022 | 2.022 | 2.056 |
| TAKTOP | 2.077 | 2.044 | 2.077 | 2.085 | 2.085 | 2.044 |
| TINRAK | 2.03 | 2.062 | 2.025 | 2.052 | 2.046 | 2.089 |
| TOKBUR | 2.037 | 2.053 | 2.037 | 2.044 | 2.044 | 2.053 |
| TUFPAM | 2.047 | 2.046 | 2.069 | 2.042 | 2.069 | 2.062 |
| TUJROG | 2.019 | 2.046 | 2.019 | 2.075 | 2.075 | 2.046 |
| UGUDIJ | 2.062 | 2.021 | 2.062 | 2.059 | 2.059 | 2.021 |
| UGUDIJ01 | 2.013 | 2.053 | 2.013 | 2.067 | 2.067 | 2.053 |
| UGUDIJ03 | 2.063 | 2.055 | 2.063 | 2.019 | 2.019 | 2.055 |
| UJEDOD | 2.062 | 2.115 | 2.062 | 2.024 | 2.024 | 2.115 |
| UJEDOD01 | 2.022 | 2.084 | 2.022 | 2.034 | 2.034 | 2.084 |
| ULAXEM | 2.056 | 2.037 | 2.056 | 2.066 | 2.066 | 2.037 |
| UMOWOJ | 2.051 | 2.002 | 2.051 | 2.072 | 2.072 | 2.002 |
| VAQDIC | 2.072 | 2.06 | 2.053 | 2.07 | 2.065 | 2.044 |
| VAQFEA | 2.055 | 2.06 | 2.051 | 2.066 | 2.068 | 2.073 |
| VELQEJ | 2.073 | 2.035 | 2.073 | 2.031 | 2.031 | 2.035 |
| VIXCEL | 2.055 | 2.039 | 2.055 | 2.039 | 2.055 | 2.055 |
| VUQVEJ | 2.032 | 2.062 | 2.032 | 2.082 | 2.082 | 2.062 |
| VUQVEJ | 2.103 | 2.054 | 2.06 | 2.018 | 2.005 | 2.039 |
| VUQZOY | 2.045 | 2.041 | 2.045 | 2.073 | 2.073 | 2.041 |
| WABMER | 2.035 | 2.063 | 2.035 | 2.044 | 2.044 | 2.063 |
| WABMER | 2.073 | 2.045 | 2.073 | 2.041 | 2.041 | 2.045 |
| WEPKUY | 2.049 | 2.043 | 2.049 | 2.062 | 2.062 | 2.043 |
| WIVCOU | 2.09 | 2.037 | 2.09 | 2.017 | 2.017 | 2.037 |
| WIVCOU | 2.005 | 2.052 | 2.052 | 2.069 | 2.076 | 2.042 |
| WOHBUQ | 2.051 | 2.079 | 2.051 | 2.056 | 2.056 | 2.079 |
| WURBUH | 2.059 | 2.085 | 2.059 | 2.053 | 2.053 | 2.085 |
| WURXUE | 2.092 | 2.057 | 2.092 | 2.032 | 2.032 | 2.057 |
| WUTGUN | 2.044 | 2.057 | 2.044 | 2.069 | 2.069 | 2.057 |
| WUZNAH | 2.062 | 2.062 | 2.062 | 2.035 | 2.035 | 2.062 |
| XATMEM | 2.028 | 2.026 | 2.047 | 2.03 | 2.099 | 2.037 |
| XEMGIF | 2.059 | 2.04 | 2.042 | 2.035 | 2.055 | 2.054 |
| XEMGIF01 | 2.057 | 2.036 | 2.04 | 2.039 | 2.053 | 2.058 |
| XEMGIF02 | 2.036 | 2.036 | 2.041 | 2.036 | 2.036 | 2.041 |
| XEMGIF04 | 2.048 | 2.031 | 2.04 | 2.037 | 2.053 | 2.055 |
| XEMGIF04 | 2.049 | 2.026 | 2.034 | 2.056 | 2.035 | 2.069 |
| XEMGIF04 | 2.035 | 2.037 | 2.049 | 2.025 | 2.041 | 2.045 |
| XEMGIF04 | 2.068 | 2.023 | 2.038 | 2.035 | 2.036 | 2.057 |
| XEMGIF04 | 2.064 | 2.043 | 2.044 | 2.034 | 2.049 | 2.058 |
| XEMGIF04 | 2.061 | 2.032 | 2.039 | 2.034 | 2.053 | 2.058 |
| XEMGIF04 | 2.063 | 2.032 | 2.042 | 2.032 | 2.042 | 2.061 |
| XEMGIF05 | 2.038 | 2.033 | 2.051 | 2.036 | 2.069 | 2.051 |
| XEMGIF05 | 2.055 | 2.042 | 2.047 | 2.031 | 2.052 | 2.064 |
| XEMGIF05 | 2.051 | 2.047 | 2.038 | 2.035 | 2.036 | 2.068 |
| XEMGIF05 | 2.054 | 2.035 | 2.048 | 2.04 | 2.053 | 2.051 |
| XEMGIF05 | 2.065 | 2.045 | 2.039 | 2.04 | 2.047 | 2.072 |
| XEMGIF05 | 2.056 | 2.026 | 2.042 | 2.033 | 2.065 | 2.054 |
| XEMGIF05 | 2.07 | 2.038 | 2.041 | 2.035 | 2.048 | 2.072 |
| XEMGIF06 | 2.056 | 2.037 | 2.039 | 2.037 | 2.054 | 2.056 |
| XEMGIF07 | 2.055 | 2.037 | 2.039 | 2.037 | 2.053 | 2.058 |
| XEMGIF08 | 2.054 | 2.036 | 2.041 | 2.037 | 2.052 | 2.055 |
| XEMGIF09 | 2.055 | 2.036 | 2.039 | 2.035 | 2.052 | 2.057 |
| XEMGIF10 | 2.054 | 2.034 | 2.039 | 2.036 | 2.053 | 2.056 |
| XEMGIF11 | 2.055 | 2.036 | 2.038 | 2.033 | 2.05 | 2.059 |
| XEMGIF12 | 2.053 | 2.037 | 2.04 | 2.034 | 2.052 | 2.058 |
| XEMGIF13 | 2.053 | 2.036 | 2.041 | 2.033 | 2.053 | 2.056 |
| XEMGIF14 | 2.054 | 2.035 | 2.041 | 2.034 | 2.052 | 2.057 |
| XEMGIF15 | 2.053 | 2.035 | 2.04 | 2.033 | 2.05 | 2.057 |
| XEMGIF16 | 2.055 | 2.033 | 2.04 | 2.033 | 2.049 | 2.056 |
| XEMGIF17 | 2.053 | 2.034 | 2.039 | 2.033 | 2.049 | 2.056 |
| XEMGIF18 | 2.053 | 2.034 | 2.039 | 2.034 | 2.049 | 2.055 |
| XEMGIF19 | 2.051 | 2.032 | 2.037 | 2.035 | 2.047 | 2.054 |
| XEMGIF20 | 2.037 | 2.037 | 2.035 | 2.024 | 2.039 | 2.039 |
| XEMGIF21 | 2.035 | 2.035 | 2.036 | 2.022 | 2.036 | 2.036 |
| XEMGIF22 | 2.035 | 2.035 | 2.032 | 2.025 | 2.037 | 2.037 |
| XEMGIF23 | 2.04 | 2.04 | 2.034 | 2.024 | 2.038 | 2.038 |
| XEMGIF24 | 2.036 | 2.036 | 2.037 | 2.024 | 2.039 | 2.039 |
| XEMGIF25 | 2.039 | 2.039 | 2.04 | 2.022 | 2.037 | 2.037 |
| XEMGIF26 | 2.046 | 2.046 | 2.043 | 2.024 | 2.045 | 2.045 |
| XEMGIF27 | 2.039 | 2.039 | 2.042 | 2.02 | 2.038 | 2.038 |
| XEMGIF28 | 2.04 | 2.04 | 2.046 | 2.04 | 2.04 | 2.046 |
| XEMGIF29 | 2.034 | 2.034 | 2.043 | 2.034 | 2.034 | 2.043 |
| YADGUH | 2.084 | 2.061 | 2.022 | 2.068 | 2.056 | 2.063 |
| YAGCIT | 2.136 | 2.098 | 2.136 | 2.117 | 2.117 | 2.098 |
| YOQGES | 2.053 | 2.03 | 2.062 | 2.038 | 2.066 | 2.061 |
| YOSCUF | 2.009 | 2.053 | 2.009 | 2.083 | 2.083 | 2.053 |
| YUDDAC | 2.05 | 2.05 | 2.05 | 2.06 | 2.06 | 2.05 |
| ZASRIU | 2.029 | 2.073 | 2.029 | 2.056 | 2.056 | 2.073 |
| ZASZOK | 2.121 | 2.056 | 2.061 | 2.108 | 2.122 | 2.099 |
| ZASZOK | 2.093 | 2.096 | 2.107 | 2.107 | 2.104 | 2.103 |
| ZERDUX | 2.053 | 2.07 | 2.053 | 2.027 | 2.027 | 2.07 |
| ZERDUX | 2.074 | 2.036 | 2.074 | 2.065 | 2.065 | 2.036 |
| ZOPGAO | 2.026 | 2.079 | 2.026 | 2.069 | 2.069 | 2.079 |
| ZZZPAW01 | 2.122 | 2.038 | 2.052 | 2.027 | 2.05 | 2.085 |
| AXOCUN | 2.02 | 2.054 | 2.02 | 2.078 | 2.078 | 2.054 |
| AYODUP | 2.048 | 2.072 | 2.048 | 2.047 | 2.047 | 2.072 |
| BAVYUS01 | 2.03 | 2.066 | 2.03 | 2.037 | 2.037 | 2.066 |
| EYARIH | 2.046 | 2.041 | 2.04 | 2.058 | 2.051 | 2.065 |
| OBUPIN | 2.05 | 2.072 | 2.05 | 2.044 | 2.044 | 2.072 |
| YAXBEH | 2.055 | 2.062 | 2.062 | 2.055 | 2.062 | 2.062 |

Example input files

Input files for Gaussian 09, using the B3LYP functional and the triple-ζ basis set 6-311G(d,p). The file does a geometry optimization, a frequency analysis and also creates molecular orbitals which can be visualized from the output file.

1. [Ni(OH_2_)_6_]^2+^, q = 2, multiplicity = 3

%chk=NiH2O_6.chk

%mem=800mb

#p b3lyp/6-311G(d,p)

opt scf=(conver=8)

gfinput POP(full,Orbitals=30)

freq

Comment Ni(II) is d8 high spin

2 3

28 0.000000000 0.000000000 0.000000000

8 0.000000000 0.000000000 2.066956333

8 0.000000000 0.000000000 -2.066956333

8 -2.066956333 0.000000000 0.000000000

8 2.066956333 0.000000000 0.000000000

8 0.000000000 2.066956333 0.000000000

8 0.000000000 -2.066956333 0.000000000

1 -0.780362667 0.000000000 2.635622000

1 0.780362667 0.000000000 2.635622000

1 -2.635622000 -0.780362667 0.000000000

1 -2.635622000 0.780362667 0.000000000

1 2.635622000 0.780362667 0.000000000

1 2.635622000 -0.780362667 0.000000000

1 0.000000000 2.635622000 0.780362667

1 0.000000000 2.635622000 -0.780362667

1 0.000000000 -2.635622000 -0.780362667

1 0.000000000 -2.635622000 0.780362667

1 0.780362667 0.000000000 -2.635622000

1 -0.780362667 0.000000000 -2.635622000

1. [Cr(OH_2_)_6_]^2+^, q = 2, multiplicity = 5 (for elongation)

%chk=CrH2O_6_elongation.chk

%mem=800mb

#p b3lyp/6-311G(d,p)

opt scf=(conver=8)

gfinput POP(full,Orbitals=30)

freq

Comment Cr is d6, Cr(II) is d4

2 5

Cr 0.000000000 0.000000000 0.000000000

O 0.000000000 2.006874000 0.000000000

O 0.000000000 -2.006874000 0.000000000

O 0.000000000 0.000000000 2.010176000

O 0.000000000 0.000000000 -2.010176000

O -2.247424000 0.000000000 0.000000000

O 2.247424000 0.000000000 0.000000000

H 0.002284000 2.569976000 0.784583000

H -0.002284000 2.569976000 -0.784583000

H 0.787499000 -0.001012000 2.569821000

H -0.787499000 0.001012000 2.569821000

H -0.787499000 -0.001012000 -2.569821000

H 0.787499000 0.001012000 -2.569821000

H -2.830627000 0.769614000 0.000409000

H -2.830627000 -0.769614000 -0.000409000

H 2.830627000 -0.769614000 0.000409000

H 2.830627000 0.769614000 -0.000409000

H 0.002284000 -2.569976000 -0.784583000

H -0.002284000 -2.569976000

1. [Cr(OH_2_)_6_]^2+^, q = 2, multiplicity = 5 (for compression)

%chk=CrH2O_6_compression.chk

%mem=800mb

#p M06/6-311G(d,p)

opt=(MaxCycle=1000) SCF=(XQC,Conver=8,MaxCycle=500) freq

scf=tight integral(grid=ultrafine) Test GFINPUT IOP(6/7=3)

Comment Cr is d6, Cr(II) is d4

2 5

24 0.000000000 0.000000000 0.000000000

8 0.000000000 2.248940000 0.000000000

8 0.000000000 -2.248940000 0.000000000

8 -2.072221000 0.000000000 0.000000000

8 2.072221000 0.000000000 0.000000000

8 0.000000000 0.000000000 -2.245662000

8 0.000000000 0.000000000 2.245662000

1 -0.773483000 2.834222000 0.000000000

1 0.773483000 2.834222000 0.000000000

1 -2.642301000 0.000000000 0.785108000

1 -2.642301000 0.000000000 -0.785108000

1 2.642301000 0.000000000 -0.785108000

1 2.642301000 0.000000000 0.785108000

1 0.000000000 0.775092000 -2.829414000

1 0.000000000 -0.775092000 -2.829414000

1 0.000000000 -0.775092000 2.829414000

1 0.000000000 0.775092000 2.829414000

1 0.773483000 -2.834222000 0.000000000

1 -0.773483000 -2.834222000 0.000000000

1. [Cu(OH_2_)_6_]^2+^, q = 2, multiplicity = 2 (for elongation)

%chk=CuH2O_6_elongation.chk

%mem=800mb

#p b3lyp/6-311G(d,p)

opt scf=(conver=8)

gfinput POP(full,Orbitals=30)

freq

Comment Cu is d11, Cu(II) is d9

2 2

Cu 0.000000000 0.000000000 0.000000000

O 0.000000000 2.006874000 0.000000000

O 0.000000000 -2.006874000 0.000000000

O 0.000000000 0.000000000 2.010176000

O 0.000000000 0.000000000 -2.010176000

O -2.247424000 0.000000000 0.000000000

O 2.247424000 0.000000000 0.000000000

H 0.002284000 2.569976000 0.784583000

H -0.002284000 2.569976000 -0.784583000

H 0.787499000 -0.001012000 2.569821000

H -0.787499000 0.001012000 2.569821000

H -0.787499000 -0.001012000 -2.569821000

H 0.787499000 0.001012000 -2.569821000

H -2.830627000 0.769614000 0.000409000

H -2.830627000 -0.769614000 -0.000409000

H 2.830627000 -0.769614000 0.000409000

H 2.830627000 0.769614000 -0.000409000

H 0.002284000 -2.569976000 -0.784583000

H -0.002284000 -2.569976000 0.784583000

1. [Cu(OH_2_)_6_]^2+^, q = 2, multiplicity = 2 (for compression)

%chk=CuH2O_6_compression.chk

%mem=800mb

#p M06/6-311G(d,p)

opt=(MaxCycle=1000) SCF=(XQC,Conver=8,MaxCycle=500) freq

scf=tight integral(grid=ultrafine) Test GFINPUT IOP(6/7=3)

Comment Cu is d11, Cu(II) is d9

2 2

29 0.000000000 0.000000000 0.000000000

8 0.000000000 2.168192000 0.000000000

8 0.000000000 -2.168192000 0.000000000

8 -1.973977000 0.000000000 0.000000000

8 1.973977000 0.000000000 0.000000000

8 0.000000000 0.000000000 -2.162175000

8 0.000000000 0.000000000 2.162175000

1 -0.776139000 2.749281000 0.000000000

1 0.776139000 2.749281000 0.000000000

1 -2.533219000 0.000000000 0.792505000

1 -2.533219000 0.000000000 -0.792505000

1 2.533219000 0.000000000 -0.792505000

1 2.533219000 0.000000000 0.792505000

1 0.000000000 0.778518000 -2.740758000

1 0.000000000 -0.778518000 -2.740758000

1 0.000000000 -0.778518000 2.740758000

1 0.000000000 0.778518000 2.740758000

1 0.776139000 -2.749281000 0.000000000

1 -0.776139000 -2.749281000 0.000000000

Optimized Cartesian coordinates (Å)

All compounds were optimized with the indicated functional, as implemented in the Gaussian 09 package^^[[8]](#endnote-8)^^, using the triple-ζ basis set 6-311G(d,p), specifying the correct charge and spin multiplicity, see example input files.

## [Ni(OH_2_)_6_]^2+^, q = 2, multiplicity = 3 (B3LYP)

Ni 0.000000000 0.000000000 0.000000000

O 0.000000000 0.000000000 2.066956000

O 0.000000000 0.000000000 -2.066956000

O 0.000000000 2.066956000 0.000000000

O 0.000000000 -2.066956000 0.000000000

O 2.066956000 0.000000000 0.000000000

O -2.066956000 0.000000000 0.000000000

H 0.000000000 0.780363000 2.635622000

H 0.000000000 -0.780363000 2.635622000

H -0.780363000 2.635622000 0.000000000

H 0.780363000 2.635622000 0.000000000

H 0.780363000 -2.635622000 0.000000000

H -0.780363000 -2.635622000 0.000000000

H 2.635622000 0.000000000 0.780363000

H 2.635622000 0.000000000 -0.780363000

H -2.635622000 0.000000000 -0.780363000

H -2.635622000 0.000000000 0.780363000

H 0.000000000 -0.780363000 -2.635622000

H 0.000000000 0.780363000 -2.635622000

## [Ni(OH_2_)_6_]^2+^, q = 2, multiplicity = 3 (M06)

Ni 0.000000000 0.000000000 0.000000000

O 0.000000000 0.000000000 2.051386000

O 0.000000000 0.000000000 -2.051386000

O 0.000000000 2.051386000 0.000000000

O 0.000000000 -2.051386000 0.000000000

O 2.051386000 0.000000000 0.000000000

O -2.051386000 0.000000000 0.000000000

H 0.000000000 0.779311000 2.617489000

H 0.000000000 -0.779311000 2.617489000

H -0.779311000 2.617489000 0.000000000

H 0.779311000 2.617489000 0.000000000

H 0.779311000 -2.617489000 0.000000000

H -0.779311000 -2.617489000 0.000000000

H 2.617489000 0.000000000 0.779311000

H 2.617489000 0.000000000 -0.779311000

H -2.617489000 0.000000000 -0.779311000

H -2.617489000 0.000000000 0.779311000

H 0.000000000 -0.779311000 -2.617489000

H 0.000000000 0.779311000 -2.617489000

## [Ni(OH_2_)_6_]^2+^, q = 2, multiplicity = 3 (OLYP)

Ni 0.000000000 0.000000000 0.000000000

O 0.000000000 0.000000000 2.099578000

O 0.000000000 0.000000000 -2.099578000

O 0.000000000 2.099578000 0.000000000

O 0.000000000 -2.099578000 0.000000000

O 2.099578000 0.000000000 0.000000000

O -2.099578000 0.000000000 0.000000000

H 0.000000000 0.778978000 2.671254000

H 0.000000000 -0.778978000 2.671254000

H -0.778978000 2.671254000 0.000000000

H 0.778978000 2.671254000 0.000000000

H 0.778978000 -2.671254000 0.000000000

H -0.778978000 -2.671254000 0.000000000

H 2.671254000 0.000000000 0.778978000

H 2.671254000 0.000000000 -0.778978000

H -2.671254000 0.000000000 -0.778978000

H -2.671254000 0.000000000 0.778978000

H 0.000000000 -0.778978000 -2.671254000

H 0.000000000 0.778978000 -2.671254000

## [Ni(OH_2_)_6_]^2+^, q = 2, multiplicity = 3 (BP86)

Ni 0.000000000 0.000000000 0.000000000

O 0.000000000 0.000000000 2.055378000

O 0.000000000 0.000000000 -2.055378000

O 0.000000000 2.055378000 0.000000000

O 0.000000000 -2.055378000 0.000000000

O 2.055378000 0.000000000 0.000000000

O -2.055378000 0.000000000 0.000000000

H 0.000000000 0.787867000 2.627548000

H 0.000000000 -0.787867000 2.627548000

H -0.787867000 2.627548000 0.000000000

H 0.787867000 2.627548000 0.000000000

H 0.787867000 -2.627548000 0.000000000

H -0.787867000 -2.627548000 0.000000000

H 2.627548000 0.000000000 0.787867000

H 2.627548000 0.000000000 -0.787867000

H -2.627548000 0.000000000 -0.787867000

H -2.627548000 0.000000000 0.787867000

H 0.000000000 -0.787867000 -2.627548000

H 0.000000000 0.787867000 -2.627548000

## [Cr(OH_2_)_6_]^2+^, q = 2, multiplicity = 5 (B3LYP, elongation)

Cr 0.000000000 0.000000000 0.000000000

O 0.000000000 2.103106000 0.000000000

O 0.000000000 -2.103106000 0.000000000

O 0.000000000 0.000000000 -2.102200000

O 0.000000000 0.000000000 2.102200000

O 2.343219000 0.000000000 0.000000000

O -2.343219000 0.000000000 0.000000000

H -0.002118000 2.675368000 -0.778279000

H 0.002118000 2.675368000 0.778279000

H -0.780879000 -0.000960000 -2.671515000

H 0.780879000 0.000960000 -2.671515000

H 0.780879000 -0.000960000 2.671515000

H -0.780879000 0.000960000 2.671515000

H 2.930590000 0.767319000 -0.000469000

H 2.930590000 -0.767319000 0.000469000

H -2.930590000 -0.767319000 -0.000469000

H -2.930590000 0.767319000 0.000469000

H -0.002118000 -2.675368000 0.778279000

H 0.002118000 -2.675368000 -0.778279000

## [Cr(OH_2_)_6_]^2+^, q = 2, multiplicity = 5 (M06, elongation)

Cr 0.000000000 0.000000000 0.000000000

O 0.000000000 0.000000000 2.074094000

O 0.000000000 0.000000000 -2.074094000

O 0.000000000 2.077986000 0.000000000

O 0.000000000 -2.077986000 0.000000000

O 2.293079000 0.000000000 0.000000000

O -2.293079000 0.000000000 0.000000000

H -0.001710000 0.777505000 2.643754000

H 0.001710000 -0.777505000 2.643754000

H -0.779941000 2.644887000 -0.000823000

H 0.779941000 2.644887000 0.000823000

H 0.779941000 -2.644887000 -0.000823000

H -0.779941000 -2.644887000 0.000823000

H 2.877599000 0.000426000 0.766118000

H 2.877599000 -0.000426000 -0.766118000

H -2.877599000 0.000426000 -0.766118000

H -2.877599000 -0.000426000 0.766118000

H -0.001710000 -0.777505000 -2.643754000

H 0.001710000 0.777505000 -2.643754000

## [Cr(OH_2_)_6_]^2+^, q = 2, multiplicity = 5 (OLYP, elongation)

Cr 0.000000000 0.000000000 0.000000000

O 0.000000000 2.124279000 0.000000000

O 0.000000000 -2.124279000 0.000000000

O 0.000000000 0.000000000 -2.121505000

O 0.000000000 0.000000000 2.121505000

O 2.424835000 0.000000000 0.000000000

O -2.424835000 0.000000000 0.000000000

H -0.001849000 2.699386000 -0.777047000

H 0.001849000 2.699386000 0.777047000

H -0.780191000 -0.000834000 -2.693119000

H 0.780191000 0.000834000 -2.693119000

H 0.780191000 -0.000834000 2.693119000

H -0.780191000 0.000834000 2.693119000

H 3.018249000 0.763832000 -0.000450000

H 3.018249000 -0.763832000 0.000450000

H -3.018249000 -0.763832000 -0.000450000

H -3.018249000 0.763832000 0.000450000

H -0.001849000 -2.699386000 0.777047000

H 0.001849000 -2.699386000 -0.777047000

## [Cr(OH_2_)_6_]^2+^, q = 2, multiplicity = 5 (BP86, elongation)

Cr 0.000000000 0.000000000 0.000000000

O 0.000000000 2.084466000 0.000000000

O 0.000000000 -2.084466000 0.000000000

O 0.000000000 0.000000000 -2.078884000

O 0.000000000 0.000000000 2.078884000

O 2.352072000 0.000000000 0.000000000

O -2.352072000 0.000000000 0.000000000

H -0.001647000 2.661509000 -0.784954000

H 0.001647000 2.661509000 0.784954000

H -0.788501000 -0.000733000 -2.651872000

H 0.788501000 0.000733000 -2.651872000

H 0.788501000 -0.000733000 2.651872000

H -0.788501000 0.000733000 2.651872000

H 2.948002000 0.771817000 -0.000379000

H 2.948002000 -0.771817000 0.000379000

H -2.948002000 -0.771817000 -0.000379000

H -2.948002000 0.771817000 0.000379000

H -0.001647000 -2.661509000 0.784954000

H 0.001647000 -2.661509000 -0.784954000

## [Cr(OH_2_)_6_]^2+^, q = 2, multiplicity = 5 (M06, compression)

Cr 0.000000000 0.000000000 0.000000000

O 2.199677000 0.000000000 0.000000000

O -2.199677000 0.000000000 0.000000000

O 0.000000000 0.000000000 2.045612000

O 0.000000000 0.000000000 -2.045612000

O 0.000000000 2.195248000 0.000000000

O 0.000000000 -2.195248000 0.000000000

H 2.779591000 0.000000000 0.769663000

H 2.779591000 0.000000000 -0.769663000

H 0.000000000 -0.781463000 2.610327000

H 0.000000000 0.781463000 2.610327000

H 0.000000000 0.781463000 -2.610327000

H 0.000000000 -0.781463000 -2.610327000

H 0.771711000 2.772820000 0.000000000

H -0.771711000 2.772820000 0.000000000

H -0.771711000 -2.772820000 0.000000000

H 0.771711000 -2.772820000 0.000000000

H -2.779591000 0.000000000 -0.769663000

H -2.779591000 0.000000000 0.769663000

## [Cu(OH_2_)_6_]^2+^, q = 2, multiplicity = 2 (B3LYP, elongation)

Cu 0.000000000 0.000000000 0.000000000

O 0.000000000 0.000000000 2.006874000

O 0.000000000 0.000000000 -2.006874000

O 0.000000000 2.010176000 0.000000000

O 0.000000000 -2.010176000 0.000000000

O 2.247424000 0.000000000 0.000000000

O -2.247424000 0.000000000 0.000000000

H -0.002284000 0.784583000 2.569976000

H 0.002284000 -0.784583000 2.569976000

H -0.787499000 2.569821000 -0.001012000

H 0.787499000 2.569821000 0.001012000

H 0.787499000 -2.569821000 -0.001012000

H -0.787499000 -2.569821000 0.001012000

H 2.830627000 0.000409000 0.769614000

H 2.830627000 -0.000409000 -0.769614000

H -2.830627000 0.000409000 -0.769614000

H -2.830627000 -0.000409000 0.769614000

H -0.002284000 -0.784583000 -2.569976000

H 0.002284000 0.784583000 -2.569976000

## [Cu(OH_2_)_6_]^2+^, q = 2, multiplicity = 2 (M06, elongation)

Cu 0.000000000 0.000000000 0.000000000

O 0.000000000 2.009256000 0.000000000

O 0.000000000 -2.009256000 0.000000000

O -2.247598000 0.000000000 0.000000000

O 2.247598000 0.000000000 0.000000000

O 0.000000000 0.000000000 -2.007648000

O 0.000000000 0.000000000 2.007648000

H -0.787623000 2.568702000 0.000000000

H 0.787623000 2.568702000 0.000000000

H -2.830642000 0.000000000 0.769705000

H -2.830642000 0.000000000 -0.769705000

H 2.830642000 0.000000000 -0.769705000

H 2.830642000 0.000000000 0.769705000

H 0.000000000 0.784570000 -2.570756000

H 0.000000000 -0.784570000 -2.570756000

H 0.000000000 -0.784570000 2.570756000

H 0.000000000 0.784570000 2.570756000

H 0.787623000 -2.568702000 0.000000000

H -0.787623000 -2.568702000 0.000000000

## [Cu(OH_2_)_6_]^2+^, q = 2, multiplicity = 2 (OLYP, elongation)

Cu 0.000000000 0.000000000 0.000000000

O 0.243769000 1.338961000 -1.519777000

O -0.243769000 -1.338961000 1.519777000

O 0.067473000 1.597188000 1.275528000

O -0.067473000 -1.597188000 -1.275528000

O -2.342829000 0.156943000 -0.089298000

O 2.342829000 -0.156943000 0.089298000

H 0.142767000 2.283361000 -1.328872000

H -0.057709000 1.203419000 -2.429702000

H 0.863946000 1.859534000 1.759092000

H -0.701688000 1.909675000 1.773424000

H -0.863946000 -1.859535000 -1.759092000

H 0.701688000 -1.909675000 -1.773424000

H -2.938469000 0.710902000 -0.611687000

H -2.930081000 -0.352921000 0.485203000

H 2.938469000 -0.710902000 0.611687000

H 2.930081000 0.352921000 -0.485203000

H -0.142767000 -2.283361000 1.328872000

H 0.057709000 -1.203419000 2.429702000

## [Cu(OH_2_)_6_]^2+^, q = 2, multiplicity = 2 (BP86, elongation)

Cu 0.000000000 0.000000000 0.000000000

O -0.252493000 -0.695185000 -1.890263000

O 0.252493000 0.695185000 1.890263000

O -0.013601000 -1.937651000 0.664836000

O 0.013601000 1.937651000 -0.664836000

O 2.339935000 -0.072093000 -0.161204000

O -2.339935000 0.072093000 0.161204000

H -0.117060000 -1.644779000 -2.071334000

H 0.019309000 -0.202470000 -2.686812000

H -0.805894000 -2.404782000 0.987003000

H 0.781999000 -2.416596000 0.960572000

H 0.805894000 2.404782000 -0.987003000

H -0.781999000 2.416596000 -0.960572000

H 2.944105000 -0.377938000 -0.860820000

H 2.920688000 0.208958000 0.568286000

H -2.944105000 0.377938000 0.860820000

H -2.920688000 -0.208958000 -0.568286000

H 0.117060000 1.644779000 2.071335000

H -0.019309000 0.202470000 2.686812000

## [Cu(OH_2_)_6_]^2+^, q = 2, multiplicity = 2 (M06, compression)

Cu 0.000000000 0.000000000 0.000000000

O -0.251358000 -0.702185000 -1.855460000

O 0.251358000 0.702185000 1.855460000

O -0.016943000 -1.911906000 0.615986000

O 0.016943000 1.911906000 -0.615986000

O 2.248909000 -0.065072000 -0.137703000

O -2.248909000 0.065072000 0.137703000

H -0.134574000 -1.656608000 -2.022278000

H -0.022812000 -0.227733000 -2.676200000

H -0.807434000 -2.370119000 0.956355000

H 0.777911000 -2.391528000 0.914222000

H 0.807434000 2.370119000 -0.956355000

H -0.777911000 2.391528000 -0.914222000

H 2.856902000 -0.361557000 -0.838751000

H 2.825009000 0.215243000 0.596293000

H -2.856902000 0.361557000 0.838751000

H -2.825009000 -0.215243000 -0.596293000

H 0.134574000 1.656608000 2.022278000

H 0.022812000 0.227733000 2.676200000

1. [] Cambridge Structural Database (CSD), Version 5.39, Feb 2018 update, Cambridge, UK, 2017. [↑](#endnote-ref-1)
2. [] C. Dobe, C. Noble, G. Carver, P.L.W. Tregenna-Piggott, G.J. McIntyre, A. Barra, A. Neels, St. Janssen, F. Juranyi, Electronic and Molecular Structure of High-Spin d^4^ Complexes:  Experimental and Theoretical Study of the [Cr(D_2_O)_6_]_2_^+^ Cation in Tutton's Salts, J. Am. Chem. Soc. 126 (2004) 16639–16652. DOI: 10.1021/ja046095c [↑](#endnote-ref-2)
3. [] B.N. Figgis, E.S. Kucharski, The structure of (ND_4_)_2_Cr(SO_4_)_2_.6D_2_O at 4.3 K by neutron diffraction, Acta Cryst. C47 (1991) 419-421. doi: 10.1107/S0108270190007776 [↑](#endnote-ref-3)
4. [] B.N. Figgis, E.S. Kucharski, P.A. Reynolds, Charge density in (NH_4_)_2_Cr(SO_4_)_2_.6H_2_O at 84 K: a Jahn–Teller distorted complex, Acta Cryst. (1990). B46, 577-586. DOI 10.1107/S0108768190005845. [↑](#endnote-ref-4)
5. [] M.A. Araya, F.A. Cotton, L.M. Daniels, L.R. Falvello, C.A. Murillo, Solid solutions of a Jahn-Teller compound in an undistorted host. 3. The chromium/zinc Tutton salt system, Inorg. Chem. 32 (1993) 4853–4860. DOI: 10.1021/ic00074a033 [↑](#endnote-ref-5)
6. [] F.A. Cotton, L.M. Daniels, C.A. Murillo, J.F. Quesada, Hexaaqua dipositive ions of the first transition series: new and accurate structures; expected and unexpected trends, Inorg. Chem. 32 (1993) 4861–4867. DOI: 10.1021/ic00074a034 [↑](#endnote-ref-6)
7. [] F.A. Cotton, L.R. Falvello, C.A. Murillo, J.F. Quesada, A completely suppressed Jahn-Teller effect in the structure of hexaaquachromium(II) hexafluorosilicate, J. Solid State Chem. 96 (1992) 192-198. DOI: 10.1016/S0022-4596(05)80311-1 [↑](#endnote-ref-7)
8. [] Frisch, M. J.; Trucks, G. W.; Schlegel, H. B.; Scuseria, G. E.; Robb, M. A.; Cheeseman, J. R.; Scalmani, G.; Barone, V.; Mennucci, B.; Petersson, G. A.; Nakatsuji, H.; Caricato, M.; Li, X.; Hratchian, H. P.; Izmaylov, A. F.; Bloino, J.; Zheng, G.; Sonnenberg, J. L.; Hada, M.; Ehara, M.; Toyota, K.; Fukuda, R.; Hasegawa, J.; Ishida, M.; Nakajima, T.; Honda, Y.; Kitao, O.; Nakai, H.; Vreven, T.; Montgomery (Jr.), J. A.; Peralta, J. E.; Ogliaro, F.; Bearpark, M.; Heyd, J. J.; Brothers, E. K; Kudin, N.; Staroverov, V. N.; Kobayashi, R.; Normand, J.; Raghavachari, K.; Rendell, A.; Burant, J. C.; Iyengar, S. S.; Tomasi, J.; Cossi, M.; Rega, N.; Millam, J .M.; Klene, M.; Knox, J. E.; Cross, J. B.; Bakken, V.; Adamo, C.; Jaramillo, J.; Gomperts, R.; Stratmann, R. E.; Yazyev, O.; Austin, A. J.; Cammi, R.; Pomelli, C.; Ochterski, J. W.; Martin, R. L.; Morokuma, K.; Zakrzewski, V. G.; Voth, G. A.; Salvador, P.; Dannenberg, J. J.; Dapprich, S.; Daniels, A. D.; Farkas, Ö.; Foresman, J. B.; Ortiz, J. V.; Cioslowski, J.; Fox, D. J. *Gaussian 09, Revision D.01*; Gaussian, Inc.: Wallingford, CT, 2009. [↑](#endnote-ref-8)
